# Decellularized vascularized bone grafts: A preliminary *in vitro* porcine model for bioengineered transplantable bone shafts

**DOI:** 10.3389/fbioe.2022.1003861

**Published:** 2023-01-18

**Authors:** Guillaume Rougier, Louis Maistriaux, Lies Fievé, Daela Xhema, Robin Evrard, Julie Manon, Raphael Olszewski, Fabien Szmytka, Nicolas Thurieau, Jean Boisson, Natacha Kadlub, Pierre Gianello, Catherine Behets, Benoît Lengelé

**Affiliations:** ^1^ Pole of Morphology (MORF)—Institute of Experimental and Clinical Research (IREC)—UCLouvain, Brussels, Belgium; ^2^ Department of Oncological and Cervicofacial Reconstructive Surgery, Otorhinolaryngology, Maxillofacial Surgery—Institut Curie, Paris, France; ^3^ Pole of Experimental Surgery and Transplantation (CHEX)—Institute of Experimental and Clinical Research (IREC)—UCLouvain, Brussels, Belgium; ^4^ Neuromusculoskeletal Lab (NMSK)—Institute of Experimental and Clinical Research (IREC)—UCLouvain, Brussels, Belgium; ^5^ Department of Maxillofacial Surgery and Stomatology—Cliniques Universitaires Saint-Luc, Brussels, Belgium; ^6^ IMSIA, ENSTA Paris, Institut Polytechnique de Paris, Palaiseau, France; ^7^ Department of Maxillofacial and Reconstructive Surgery—Necker Enfants Malades, Paris, France; ^8^ Department of Plastic and Reconstructive Surgery—Cliniques Universitaires Saint-Luc, Brussels, Belgium

**Keywords:** perfusion decellularization, bone allografts, adipose mesenchymal stem cells, ECM, extracellular matrix, bone reconstruction engineering, tissue engineering, regenerative medecine

## Abstract

**Introduction**: Durable reconstruction of critical size bone defects is still a surgical challenge despite the availability of numerous autologous and substitute bone options. In this paper, we have investigated the possibility of creating a living bone allograft, using the perfusion/decellularization/recellularization (PDR) technique, which was applied to an original model of vascularized porcine bone graft.

**Materials and Methods**: 11 porcine bone forelimbs, including radius and ulna, were harvested along with their vasculature including the interosseous artery and then decellularized using a sequential detergent perfusion protocol. Cellular clearance, vasculature, extracellular matrix (ECM), and preservation of biomechanical properties were evaluated. The cytocompatibility and *in vitro* osteoinductive potential of acellular extracellular matrix were studied by static seeding of NIH-3T3 cells and porcine adipose mesenchymal stem cells (pAMSC), respectively.

**Results**: The vascularized bone grafts were successfully decellularized, with an excellent preservation of the 3D morphology and ECM microarchitecture. Measurements of DNA and ECM components revealed complete cellular clearance and preservation of ECM’s major proteins. Bone mineral density (BMD) acquisitions revealed a slight, yet non-significant, decrease after decellularization, while biomechanical testing was unmodified. Cone beam computed tomography (CBCT) acquisitions after vascular injection of barium sulphate confirmed the preservation of the vascular network throughout the whole graft. The non-toxicity of the scaffold was proven by the very low amount of residual sodium dodecyl sulfate (SDS) in the ECM and confirmed by the high live/dead ratio of fibroblasts seeded on periosteum and bone ECM-grafts after 3, 7, and 16 days of culture. Moreover, cell proliferation tests showed a significant multiplication of seeded cell populations at the same endpoints. Lastly, the differentiation study using pAMSC confirmed the ECM graft’s potential to promote osteogenic differentiation. An osteoid-like deposition occurred when pAMSC were cultured on bone ECM in both proliferative and osteogenic differentiation media.

**Conclusion**: Fully decellularized bone grafts can be obtained by perfusion decellularization, thereby preserving ECM architecture and their vascular network, while promoting cell growth and differentiation. These vascularized decellularized bone shaft allografts thus present a true potential for future *in vivo* reimplantation. Therefore, they may offer new perspectives for repairing large bone defects and for bone tissue engineering.

## 1 Introduction

Large bone defects usually result from traumatic losses of substance, surgical resection of primary or secondary bone tumors, sepsis, or corrective surgeries of orthopedic deformities (Anract et al., 2014; Roddy et al., 2018). Reconstructions remain a challenge despite the availability of various technical options and bone substitutes in current surgical practice (Balogh et al., 2012). Indeed, these reconstructions can be handled in different ways, depending on location, etiology, and patient’s condition, as follows: conventional techniques including autologous bone grafts (Sun et al., 2019), prosthetic surgery (Ogura et al., 2018; Jamshidi et al., 2020), peri-implant membrane induction with secondary grafting (Masquelet and Begue, 2010; Giannoudis et al., 2016; Sivakumar et al., 2016), reconstructive microsurgery (Pederson and Grome, 2019; Bibbo, 2021), distraction osteogenesis (Lesensky and Prince, 2017), or a combination of these (Niu et al., 2011; Houben et al., 2019; Kang et al., 2020). However, all procedures are associated with several disadvantages. Indeed, autologous bone grafts and microsurgical transfers, as well, induce donor site morbidity (Mauffrey et al., 2016; Morelli et al., 2016; Sparks et al., 2017), whereas large prosthetic implants are burdened with either infections or implant failure (Anract et al., 2014). Banked human bone allografts that are being used after a decellularization step or without a banking processing are currently employed for reconstructing small or large bone defects, thereby preserving a near-perfect biomechanical macro- and micro-architecture (Delloye et al., 2007). However, many studies have highlighted an imbalance of the creeping substitution of these allografts with inherent complications on account of the lack of proper vasculature, e.g., stress fracture, non-union, infection, or resorption (Delloye et al., 2007b; Delloye and Cornu, 2003). Though the addition of osteo-inductive factors or stem cells to promote their osteointegration is likely promising, clinical evidence of their usefulness is still lacking (Kasten et al., 2008; Dumic-Cule et al., 2015; García-Gareta et al., 2015). Therefore, bone substitutes and biomaterials have undergone numerous investigations, representing one of the most explored and challenging fields in tissue engineering (Delloye et al., 2003; Tang et al., 2016; Wang et al., 2018; Zhang Hao et al., 2019; Abdollahiyan et al., 2020). These materials constitute a major strategic option regardless of their drastic requirements, including the absence of immune rejection, perfect biocompatibility, close mechanical properties, as well as their ability to develop neo-angiogenesis, the latter being essential to lasting osteointegration.

The ideal substitute must be easy to use and handle, and be produced at a moderate cost, while displaying a structure comparable with a mineralized extracellular matrix (ECM) including osteoinductive factors and structural components. Moreover, recent works on periosteal regeneration and soft tissue reconstruction around large skeletal defects have highlighted that living tissues surrounding the bone do indeed play a prominent role in osteointegration (Masquelet and Begue, 2010; Baldwin et al., 2017). Recent publications regarding bioprinted bone-like materials with osteoconductive hydrogels (Anada et al., 2019) have raised great hopes in view of the restorative potentials of 3D-printed bone substitutes. But until now, they have failed to achieve a large enough size with an anatomic design and adequate microstructure to be successfully implanted under critical clinical conditions (Arealis and Nikolaou, 2015; Ravnic et al., 2017; Vidal et al., 2020). The key to long-lasting osteointegration is an immediate functional vasculature within the skeletal substitute, which cannot be obtained outside the context of a conventional organ transplant (Wang et al., 2018; Zhang et al., 2018; Zhang Hualin et al., 2019). The input of an intrinsic vasculature providing optimal blood supply to an entire bone shaft still remains the ultimate challenge to solve. Moreover, recent reports have demonstrated that tissue engineering techniques, such as the perfusion/decellularization/recellularization (PDR) process, allow the generation of 3D acellular ECM scaffolds with a preserved vascular tree. These acellular matrices can then be seeded with specific autologous cells in an effort to regenerate a functional, biocompatible, and transplantable graft (Orlando et al., 2013). These processes were first described in the organ field (Ott et al., 2010, 2008; Uygun et al., 2010; Orlando et al., 2012; Mirmalek-Sani et al., 2013), and they were later applied to a wide range of animal and human vascular composite tissues or anatomical subunits (Jank et al., 2017, 2015; Duisit et al., 2018b; 2018a, 2017; Gerli et al., 2018; Moser et al., 2020; Wüthrich et al., 2020). Although decellularization of non-vascularized bone allografts has been widely described in tissue engineering or clinical biobank (Blaudez et al., 2020), this perfusion strategy has so far never been applied to entire long bones.

In this report, we have described the first large animal model of perfusion-decellularized bone graft, which was harvested from the porcine forearm and vascularized by the interosseous pedicle. Cell clearance, ECM, and vascular tree preservation, as well as the mechanical properties of decellularized bone grafts, were evaluated. Lastly, acellular periosteum and bone ECM were seeded with NIH-3T3 cells, and the osteoinductive properties of the acellular bone ECM were studied after static seeding of porcine adipose mesenchymal stem cells (pAMSC).

## 2 Materials and methods

All experiments were approved by the local ethics committee of UCLouvain (Brussels, Belgium) and carried out in accordance with the Belgian (Royal Decree, September 2004) and European legislations (Directive-2010-63 UE) concerning animals used in experiments.

### 2.1 Harvesting technique

**FIGURE 1:**
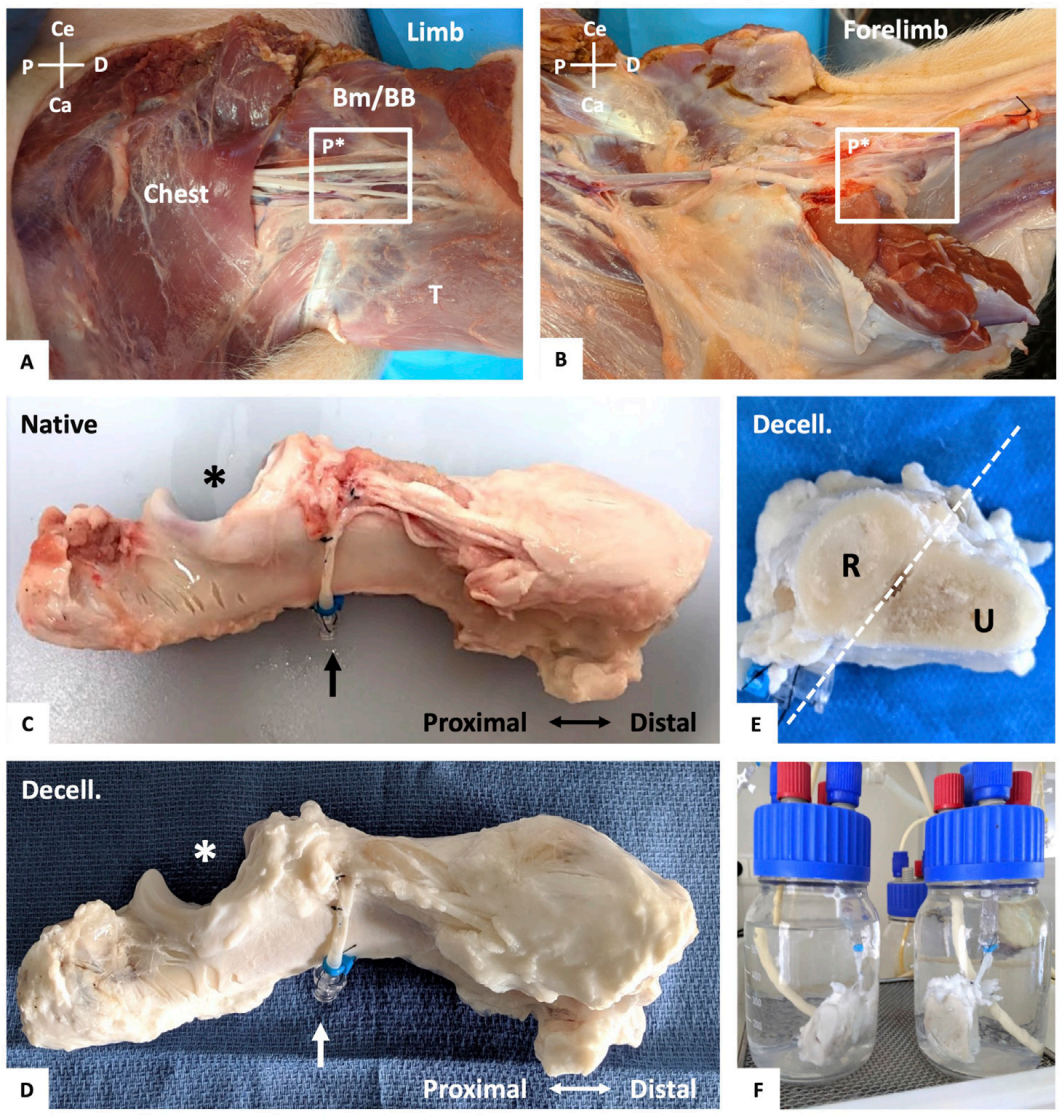
Vascularized forelimb bone graft harvesting and perfusion decellularization process. **(A, B)**: Dissection of the left brachial vascular pedicle (P*) between the chest muscles, triceps (T), biceps brachii (BB) and brachialis muscle (Bm) with identification of the vascular pedicle **(A)**. Transection of the limb and forelimb muscles and identification of the inter-osseous foramen and its vascular pedicle (P*) **(B)**. For **(A)** and **(B)**, both are anterior views with: Ce = cephalic, Ca = caudal, P = proximal, and D = distal. **(C)** Native vascularized forelimb bone graft following muscle excision, with its preserved vascular pedicle (artery and vein, black arrow) and ulnar notch (*). **(D)** Final aspect of decellularized forelimb bone graft after perfusion decellularization process with its preserved vascular pedicle (white arrow). **(E)** Axial section of the decellularized forelimb **(C)** with the radius (R) and ulna (U). **(F)** Example of potentially segmented decellularized bone graft in perfusion setup.

Porcine forelimbs were harvested from 11 female Landrace pigs aged between 6 and 10 months old (mean weight: 85.2 kg) that were used for experiments by another research group in the laboratory, after euthanasia by potassium chloride (KCl) injection. The direct approach of the forelimb vasculature between the chest muscles and Serratus muscular fasciae was employed (Figure 1A). Thoraco-dorsal and axillary vessels were dissected and isolated after transection of the surrounding nerves. Dissection of the axillary vessels was then continued until the elbow joint was reached, and the inter-osseous artery foramen was landmarked after transection of the brachioradialis muscle and exposure of the periosteum (Figure 1B). The interosseous vessel’s origin was preserved, and the pedicle was dissected until the wrist joint, and it was then ligated. The grafts were harvested after proximal and distal disarticulation, including both the radius and ulna, along with a cuff of surrounding soft tissues. A 16-G Luer needle was inserted into the artery (Figure 1C). Thereafter, the grafts were flushed with heparinized saline, while arterial leakages were sutured using 9.0 Dafilon sutures (Braun, SW). Lastly, the grafts were connected to a Masterflex L/S peristaltic pump (Cole-Palmer, Vernon Hills, IL, United States) and perfused at 12 ml/min with 1 L of cold saline serum containing 50 UI/ml of heparin (B. Braun Medical SA, Belgium) and 10 µM of adenosine (A-4036, Sigma-Aldrich).

### 2.2 Perfusion-decellularization protocol and tissue sampling

Immediately after the procurement, the bone grafts were decellularized, at room temperature, using a sequential perfusion of detergents through the vascular pedicle according to our previously published protocol (Duisit et al., 2018b; 2018a, 2017), with a constant flow rate of 12 ml/min: (1) 70 L of 1% sodium dodecyl sulfate (SDS) (27926.295, VWR) followed by 3 L of phosphate-buffered saline (PBS); (2) 40 L of 1% Triton X-100 (M143, VWR) with subsequent agitation (250rpm) overnight inside a glass jar filled with 1% Triton X-100, followed by an arterial perfusion of 40 L of PBS. Thereafter, grafts were perfused at 4 ml/min with 1 L of Type I DNAse from bovine pancreas (11284932001, Roche, Sigma-Aldrich) at 37°C (3), and then washed with 3 L of PBS (4). Grafts were stored in PBS at 4°C. Native and decellularized grafts were each sampled in muscular, periosteal, cortical bone, and medulla samples taken in proximal, central, and distal locations from the vascular pedicle for histology. For DNA, ECM proteins, and SDS quantification, biopsies were processed immediately after the harvest for native tissues and within days after the decellularization end. They were then frozen at −20°C until use.

### 2.3 Histology

Native and decellularized (n = 5) muscle or periosteal samples were fixed in 4% formalin. Next, they were embedded in paraffin, sectioned into 5 µm-thick slices, and stained before mounting. The same protocol was applied to monobloc or cortical and medulla biopsies after 3 weeks of decalcification in daily-changed baths of decalcifying solution (formic acid, 28% formalin, and deionized water). Hematoxylin eosin (H&E), Masson’s trichrome (MT), and Sirius red (SR) staining were performed. The slices were digitized and analyzed using a slide scanner (SCN400, Leica Microsystems). The 4′,6-diamidino-2-phenylindole (DAPI) staining was conducted and visualized using fluorescence microscopy (AxioImager Z1, Zeiss). Concerning immunohistochemistry (IHC), after deparaffinization, endogenous peroxidases were inhibited with 3% hydrogen peroxide in methanol. Non-specific binding sites were blocked with 5% BSA in 0.05% Triton in Tris-buffered saline. Sections were then incubated at 4 °C overnight with anti-GFP (1:5,000, MA5-15349, ThermoFischer Scientific) and anti-osteocalcin (1:100, MA1-20786, ThermoFischer Scientific) primary antibodies followed by peroxidase-conjugated anti-mouse secondary antibodies (K4001, Dako) or anti-mouse-HRP (715-035-151, Jackson ImmunoResearch, Bioconnect). They were revealed with 3.3′-diaminobenzidine (DAB) peroxidase substrate (K3468, Dako). Nuclei were counterstained with hematoxylin, and slides were mounted with Entellan New (1079610100, Merck, Sigma-Aldrich).

### 2.4 DNA and ECM proteins measurements

Biopsies from native (n = 5) and decellularized (n = 5) periosteum, muscles, medulla, and cortical bones were processed after freeze-drying. DNA was extracted, using DNEasy Blood and Tissue kit (Qiagen, Hilden, Germany), from 25 mg wet biopsies and quantified using the quant-it Picogreen dsDNA Reagents kit (L3224, ThermoFischer Scientific). GAGs and collagen proteins, using 25 mg and 20 mg wet biopsies respectively, were quantified based on the Blyscan sulfated glycosaminoglycan assay kit (Biocolor Ltd., Carrickfergus, UK) and Total Collagen Assay kit (QuickZyme, Biosciences, The Hague, Netherlands), respectively, according to each manufacturer’s protocol. Mean DNA amount was expressed in ng/mg dry weight ± SD; mean collagen and mean GAGs amount were expressed in μg/mg dry weight ± SD.

### 2.5 SDS quantification

The residual SDS in muscle, periosteum, and bone ECM (n = 3 for each tissue) was quantified using the methylene blue active substance assay (MBAS) according to a previously published protocol (Andrée et al., 2014). Briefly, 60 mg biopsies were carried out and freeze-dried. A standard curve was conducted in order to calculate the SDS amount: 1 μl of SDS 0.5%–0.25%–0.125%–0.0625%–0.0313%–0.01565%–0.0078%–0.0039% and 0% (deionized water - DIW) was mixed with 249 μl of DIW; they were then processed like the samples. The dried matrix was weighed and incubated overnight in a solution of proteinase K (1.07393.0010, Merck, Sigma-Aldrich) (10 μl Proteinase K—19.1 mg/ml - in 30 mM Tris, pH 8.0) at 50°C. Then, 250 μl of samples or standards were mixed with 250 μl of methylene blue and vortexed. Then, 500 μl chloroform (1.02445, VWR) was added to each sample or standards and vortexed. Finally, 200 μl of the chloroform layer were placed in a 96-well plate; it was measured at 651 nm using a microplate reader (Spectramax I3). The residual SDS in ECM was calculated based on the standard curve and expressed in μg/mg dry weight ± SD for SDS residues in the ECM and in μg/mL ± SD for SDS concentration in the digested proteinase K solution.

### 2.6 Vasculature evaluation

To assess the vascular tree preservation and if the radius or ulna could be harvested independently, native and decellularized grafts were arterially injected right after the harvest for the native grafts, and right after the decellularization process for the decellularized grafts, using a solution of latex mixed with barium sulphate (243353, Sigma-Aldrich) and red dye. They were kept overnight at 4°C, and then imaged (n = 3) with cone beam computed tomography (CBCT) (Planmeca Promax 3D Mid, Helsinki, FI). DICOM images were analyzed and 3D-reconstructed using Osirix software (Pixmeo, Bernex, SW).

### 2.7 Bone mineral density measurements and pQCT acquisitions

Bone mineral density (BMD; mg of hydroxyapatite/cm^3^) of ulna and radius was assessed using peripheral quantitative computed tomography (pQCT, XCT 540 Stratec SA+, Norland Stratec, Germany) within 3 days before (native, n = 3 each) decellularization and within 5 days after decellularization end (n = 3 each). In each bone, 30 slices spaced 0.1 mm apart were acquired, and 10 BMD measures were carried out in distal, central, and proximal locations from the vascular pedicle respectively. The values of the 30 slices were averaged for each bone, before and after decellularization. Data were expressed as mean BMD ±SD.

### 2.8 Mechanical testing

Multiple similarly shaped cortical bone rods were harvested from native and decellularized bones (for each: n = 3 ulna; n = 3 radius), preserved in PBS at 4°C, and tested within 5 days after the harvest (native samples) or the decellularization end (decellularized samples). The 3-pt bending tests (Figure 6A) were performed at a speed of 2 mm min^−1^ until fracture, using an automatic Instron^®^ testing machine (Instron 5,967, Instron^®^, Division of ITW Limited, Coronation Road, High Wycombe, Bucks HP12 3SY), with results compared. The test was considered successful and ended if a fracture occurred (Figure 6A). A curve was conducted while plotting the applied force (Newtons, N) *versus* the vertical displacement (mm) and results were expressed as the mean value to occur a fracture in N/mm ± SD. In addition, four cortical bone samples (n = 3 native, n = 3 decellularized) that were harvested on both the radius and ulna in each location (central, distal, and proximal) were preserved in PBS at 4°C, and tested, again also within 5 days after the harvest (native samples) or the decellularization end (decellularized samples), using a hardness-testing (Figure 6D) automatic Fischer machine (Microduromètre Fischerscope HM 2000, Fischer Technology Inc.750 Marshall Phelps Rd. CT 06095 Windsor, United States). Overall, 10 hardness measurements were performed on cortical samples (Figure 6D) using a 2000 mN load, an increase and decrease time of 20 s, and a 5 s peak time. Results were expressed as the mean Hardness Value (HV) in Vickers unit ±SD.

### 2.9 Sterilization and cyto-compatibility assay of decellularized bone ECM

Periosteum or bone ECM patches of 7 mm × 7 mm were sterilized by means of bath agitation in 0.1% peracetic acid (PAA) and 4% ethanol solution overnight for periosteum and 6 days for bone discs, with a solution renewal each day. It was followed by several baths of DIW and PBS supplemented with an antibiotic consisting of 1% penicillin/streptomycin (P/S, 15140122, ThermoFischer Scientific), 10 μg/ml gentamicin (G1397, Sigma-Aldrich), and 2.5 μg/ml amphotericin B (15290-026, ThermoFischer Scientific). ECM patches placed in 48-well plates were incubated overnight with Dulbecco’s Modified Eagle Medium (DMEM, 733-1,698, Lonza, Westburg, Netherlands); they were supplemented with 10% of Fetal Bovine Serum (FBS, 10270-106, ThermoFischer Scientific) and antibiotics. After medium removal, 75.000 NIH3-T3 cells (93061524, Sigma-Aldrich) in 10 µl were seeded on ECM discs or control wells; they were then incubated for 2 h in a cell culture incubator (37°C; 5% CO_2_) before adding 1 ml of medium that was changed every 2 days. PrestoBlue Cells Viability Assay (A13262, ThermoFischer Scientific) was carried out in order to assess the cell proliferation at Days 1, 3, 5, 7, and 16 on six seeded periostea (n = 3 donors). Culture medium was removed and replaced by 0.1% PrestoBlue solution and then incubated for 1 h. Then, 100 μl of supernatant was transferred to a 96-well plate. The fluorescent signal was measured using a microplate fluorometer (Spectramax I3^®^) at 560/590 nm, with results expressed in fluorescence intensity ±SD. H&E and Live/Dead staining (L-3224, Life Technologies, ThermoFischer Scientific) were performed at Days 3, 7, and 16 (n = 3) in order to evaluate the presence of viable cells on seeded bone and periosteum ECM. The viability percentage (%) at Day 7 on seeded periosteum and control wells was evaluated by quantifying the ratio between the green area (live cells) and addition of the green and red (dead cells) areas, after removing the artefacts, which was expressed in percentages. The latter was quantified on four different pictures taken at 2.5x magnification using a fluorescence microscope (AxioImager Z1, Zeiss), which were analysed using the FIJI^®^ software.

### 2.10 pAMSC cell seeding culture and bone-like ECM differentiation

Porcine GFP-pAMSC were graciously provided by the experimental surgery and transplantation laboratory (Prof. Gianello, CHEX, UCLouvain, Brussels, Belgium). Cells were isolated and cultured as previously published (Schubert et al., 2011). Approximately 5 × 10^5^ cells were seeded on each decellularized bone ECM under two conditions (n = 3 for each) including: 1) bone ECM + proliferation medium (PM = DMEM containing, 2 mM l-glutamine and supplemented with 1% P/S and 2.5 μg/ml of amphotericin-B 1 ml/ml); 2) bone ECM + differentiation medium (DM = PM + 1 mM dexamethasone (D4902, Sigma-Aldrich), 50 ng/ml of sodium ascorbate (A4034, VWR), and 36 mg/ml of sodium dihydrogen phosphate monohydrate (1.06346, Sigma-Aldrich)). As a control, pAMSC were seeded in a 6-well plate and cultured with either PM or DM. Culture plates were placed in a cell culture incubator for 2 h, following which PM was added in each well. After 2 days of culture, PM was changed for Group 1, with differentiation medium added to Group 2. The culture medium was changed every 2 days until osteo-differentiation was visible and could be assessed. Culture plates were observed each day during the osteogenic differentiation using an optic microscope. After 22 days of culture, samples were formalin-fixed and paraffined for standard histology, as well as anti-osteocalcin and anti-GFP IHC staining. Alizarin red staining was carried out at Day 7 on the control well to assess the differentiation with DM; at Day 22, it was similarly performed on both groups in order to evaluate pAMSC osteogenic differentiation.

### 2.11 Statistical analyses

All statistical analyses were performed using GraphPad Prism Version 8 (GraphPad Software, San Diego, United States of America). All data were expressed as mean ± standard deviation (SD). Normality was verified using Shapiro-Wilk test, with specific unpaired t-tests applied thereafter. For all tests, statistical significance was at *p* < 0.05.

## 3 Results

### 3.1 Vascularized bone graft harvesting and decellularization process

Overall, 22 forearm skeletons were successfully harvested from 11 pigs, following meticulous dissection of their vascular pedicle, while fully preserving their intrinsic vasculature (Figures 1A–C). Macroscopically, from the beginning of the arterial heparinized serum perfusion, we observed a dark venous return that progressively became clearer. During SDS perfusion, a gradual whitening of the muscle and periosteum was noticed without any alteration to their ultrastructure. At the decellularization perfusion end, all the specific tissues of the vascularized bone grafts appeared uniformly white, with their native 3D morphology fully preserved (Figures 1C–E) and can be segmented in a vascularized acellular diaphysal bone graft (Figure 1F).

### 3.2 Decellularization efficiency

**FIGURE 2:**
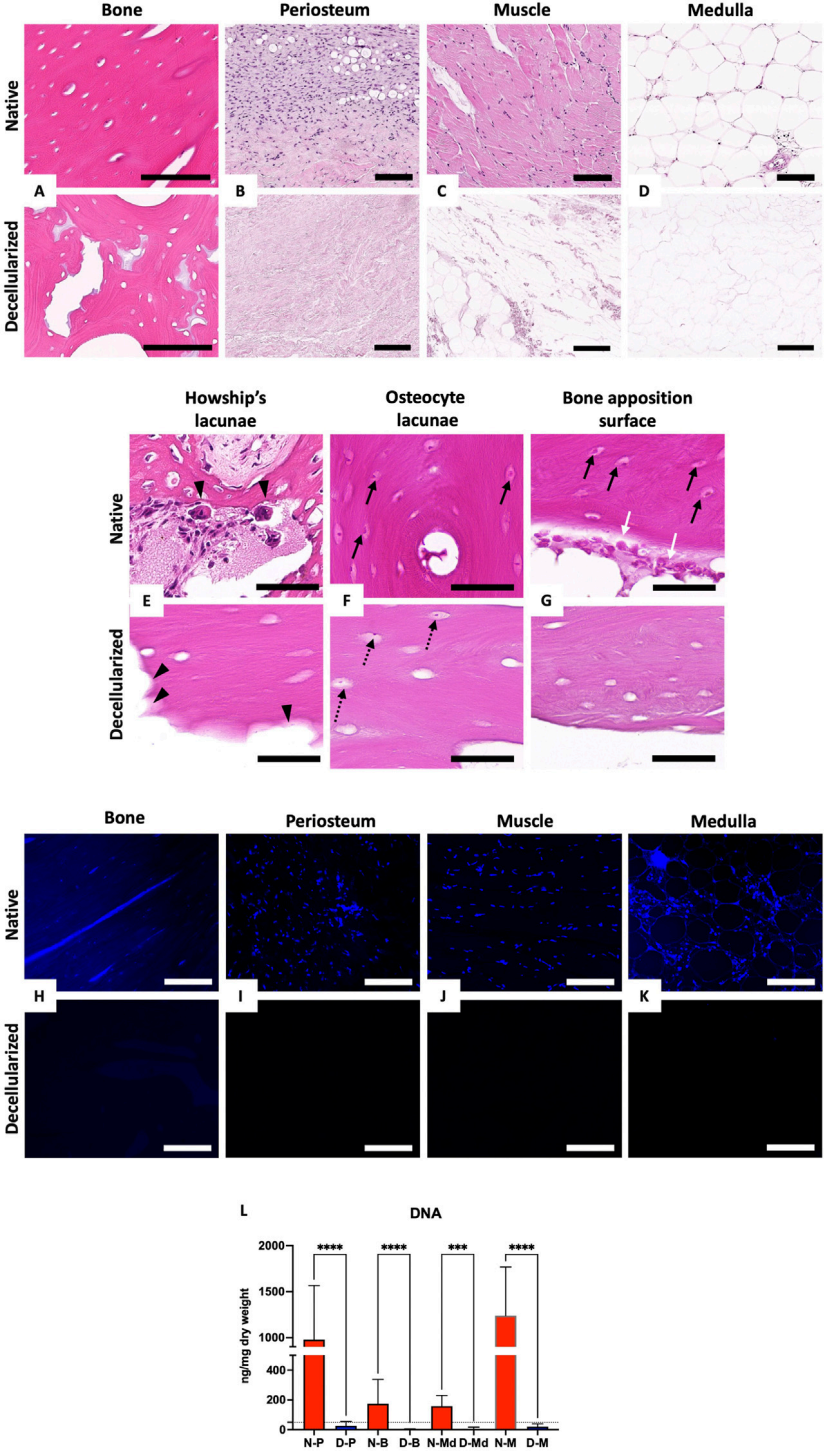
Evaluation of decellularization efficiency. **(A–G)**: H&E histological staining of native (top) and decellularized (bottom) cortical bone **(A)**, periosteum **(B)**, muscle **(C)** and medulla **(D)**. Magnification of native (top) and decellularized (bottom) bone resorption lacunae (Howship’s lacunae) **(E),** osteocyte lacunae **(F)** and bone apposition surface **(G)**; head arrows = Howship’s lacuna, white arrows = osteoblasts, black arrows = native osteocytes, dotted arrows = residual cells. Scale bar for **(A)** = 100 μm, **(B–D)** = 200 µm and for **(E–G)** = 50 µm. **(H–K)**: DAPI staining of native (top) and decellularized (bottom) cortical bone **(G)**, periosteum **(H)**, muscle **(I)** and medulla **(J)**. All scale bars = 100 µm. **(L)**: DNA quantification in native (N-, red) and decellularized (D-, blue) muscle (M), cortical bone (B), periosteum (P), and medulla (Md) (n = 5 each, mean values expressed in ng/mg dry weight ± SD; ***p < 0.001, ****p < 0.0001).

H&E and DAPI staining showed the cellular clearance in cortical bone, periosteum, muscle, and medulla samples in decellularized grafts (Figures 2A–K). We also noticed the complete removal of osteoblasts and osteoclasts from bone apposition surfaces and Howship’s resorption lacunae, respectively. However, in some histological sections, we detected, into the osteocyte lacunae of cortical or cancellous bone areas, the presence of residual cell debris and nuclei which appeared punctiform, smaller than in native lacunae (Figure 2F). Some cellular debris were also visible into the medulla. DNA quantification confirmed these findings, revealing a major and significant decrease in DNA amount, with a mean 95% reduction in decellularized tissues in comparison with native tissues. The DNA amount, expressed in ng/mg dry weight ±SD (n = 5) found in decellularized muscle, periosteum, medulla and cortical bone was 19.79 ± 19.83, 26.71 ± 28.26, 1.28 ± 4.43, and 5.78 ± 11.99, respectively, compared with 1,020.62 ± 528, 977.7 ± 587.9, 173.6 ± 163.7, and 158.7 ± 71.29 in the native samples, which was under the critical level of 50 ng/mg dry weight (Crapo et al., 2011) (Figure 2).

### 3.3 Extracellular matrix preservation

**FIGURE 3:**
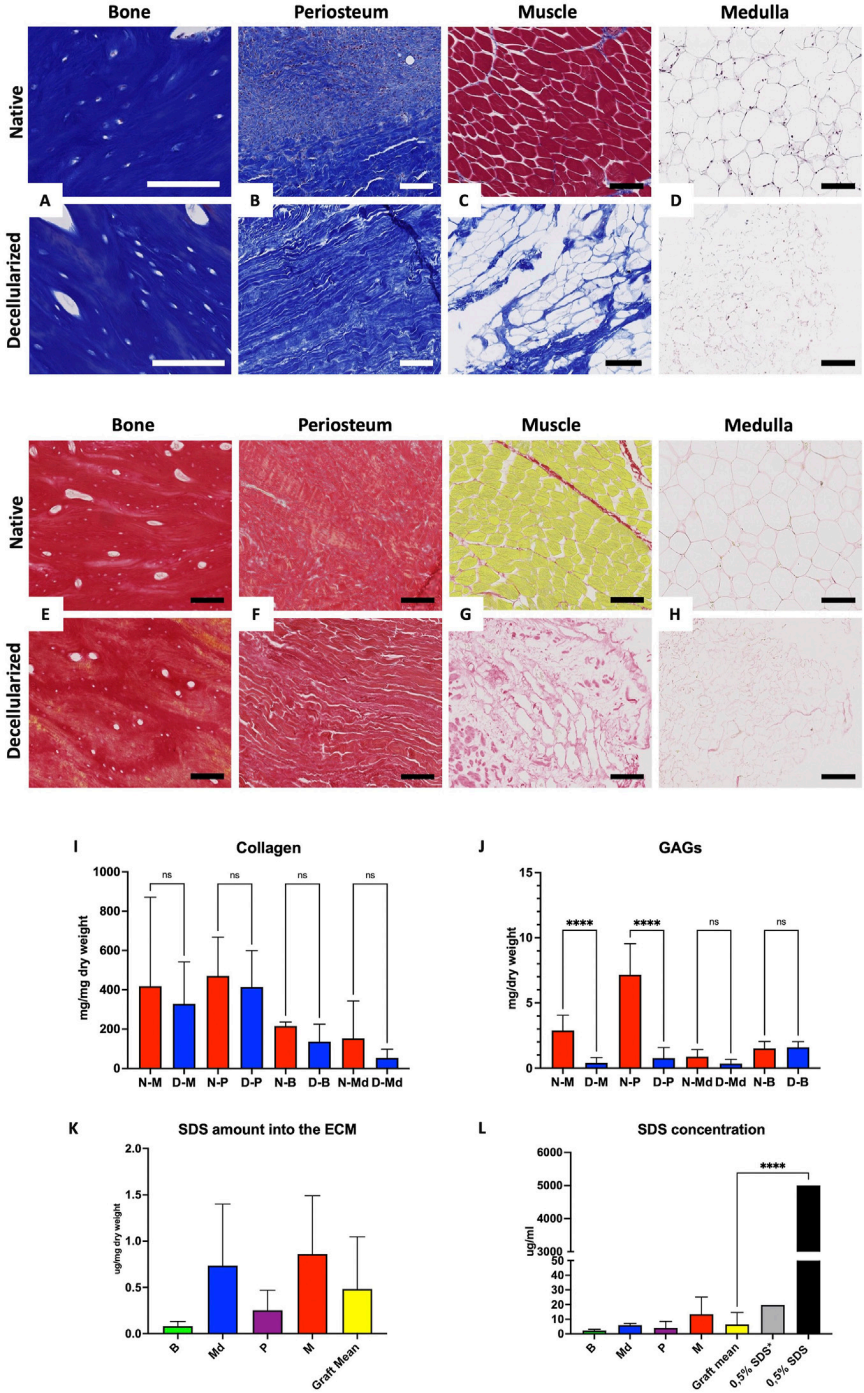
Preservation of the ECM and residual SDS quantification. **A–D:** Masson’s trichrome histological staining of native (top) and decellularized (bottom) cortical bone **(A)**, periosteum **(B)**, muscle **(C),** and medulla **(D)**. Scale bar for **(A)** = 100 µm and B-C-D = 200 µm. **(E–H)**: Sirius red histological staining showing collagen fibers, preservation of native (top) and decellularized (bottom) cortical bone **(E)**, periosteum **(F)**, muscle **(G),** and medulla **(H)**. All scale bars 100 µm. **(I–J)**: Collagen **(I)** and GAGs **(J)** quantification in native (N-, red) and decellularized (D-, blue) muscle (M), cortical bone (B), periosteum (P), and medulla (Md) (n = 5 each, mean values expressed in µg/mg dry weight ± SD; *ns* = non-significant, ****p < 0.0001). **(K–L)**: SDS residues into acellular ECM **(K)** and SDS concentration in digested tissues with 1 ml of proteinase K solution **(L)** in decellularized cortical bone (B), medulla (Md), periosteum (P), and muscle (M) in comparison with 0.5% SDS* (0.5% SDS diluted 250×) and theorical 0.5% SDS solution (n = 3 each, mean values expressed in µg/ml **(K)** and µg/mg **(L)** dry weight ± SD; ****p < 0.0001).

H&E, MT, and SR staining confirmed the preservation of ECM and tissue microscopic architecture as well as collagen fibers stained by SR for the cortical bone (CB), periosteum (P), muscle (M), and medulla (Md) samples in decellularized grafts (Figures 3A–H). Collagen analysis revealed a non-significant difference between native (Nat) and decellularized (Decell.) samples (Decell. vs Nat: M: 328.8 ± 213.3 vs 417.1 ± 454.1; P: 413.784 ± 185.2 vs 468.0 ± 194.0; Md: 53.2 ± 43.98 vs 151.2 ± 191.6, CB: 136.7 ± 88.21 vs 205.1 ± 41.95 μg/mg dry weight ±SD, n = 5) (Figure 3I). These quantitative data confirmed the SR and MT staining findings. Both methods revealed the preservation of the ECM architecture, and especially collagen fibers and tissular collagen content, without perceptible modifications of the histological and tissular architecture. In addition, GAG measurements revealed non-significant variations for cortical bone and medulla (Decell. vs Nat: CB: 1.6 ± 0.2 vs 1.0 ± 0.52; Md: 0.3543 ± 0.1 vs 0.8 ± 0.4 μg/mg dry weight ± SD, n = 5), except for the muscle and periosteum, both showing a significant decrease between native and decellularized samples (Decell. vs Nat: M: 0.4073 ± 0.39 vs 2.890 ± 1.16; P: 0.76 ± 0.81 vs 7.164 ± 2.37 μg/mg dry weight ± SD, n = 5) (Figure 3J).

### 3.4 SDS residues in ECM quantification

SDS is a strong cytotoxic decellularization detergent. The MBAS was used to detect the residual SDS linked to ECM after its release following the digestion of the dried ECM by a proteinase K solution. The SDS amount in the ECM was low, being comparable to the results found by other investigators (Andrée et al., 2014). Residual SDS in tissue samples (µg/mg dry weight ± SD, n = 3) was recorded as follows: M: 0.86 ± 0.62; P: 0.25 ± 0.21; Md: 0.73 ± 0.66; B: 0.081 ± 0.05, with the average residual amount for the entire graft being 0.48 ± 0.56 (Figure 3K). The SDS concentration (µg/mL ± SD, n = 3) found in the digested solution from all tissues was significantly lower than the perfused SDS solution, corresponding to M: 13.44 ± 11.67; P: 4.13 ± 4.40; Md: 5.92 ± 1.27; B: 2.25 ± 0.83, while the entire graft retained 6.52 ± 8.09 of SDS (Figure 3L). Based on this observation, SDS-based decellularized matrices can be efficiently washed out with DIW and PBS.

### 3.5 Vasculature anatomical study

**FIGURE 4:**
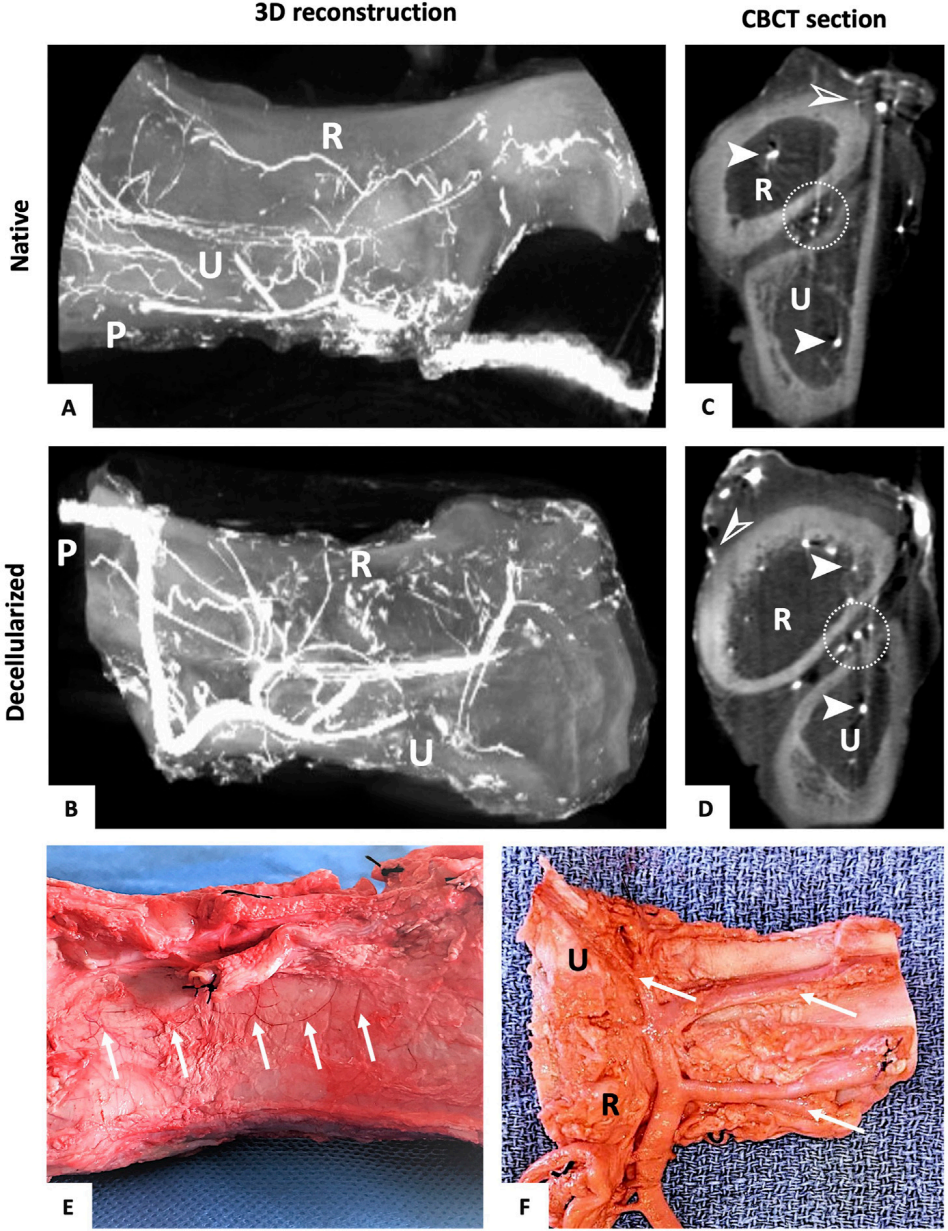
Vascularization of the vascularized bone graft. **(A–F)**: 3D reconstruction of the forelimb graft before **(A)** and after decellularization **(B)** showing the vascular tree preservation (R = radius, U = ulna, *p* = pedicle). CBCT transversal section of barium sulphate injected into the forelimb bone graft before **(C)** and after perfusion decellularization **(D)** (R = radius, U = ulna) showing the perfusion of barium sulphate into endomedular vessels (➤), interosseous vessels (white circle), and subperiosteal vessels (➢) before and after decellularization. Identification of the preserved periosteal vascular network (white arrows) **(E)** and intra-osseous perforators (white arrows) **(F)** after dissecting a latex-injected decellularized bone graft (R = radius, U = ulna).

CBCT acquisitions of decellularized bone grafts after intra-arterial contrast injection (n = 3) confirmed the preservation of the vascular network arising from the main interosseous artery while giving off numerous intraosseous vessels and periosteal ramifications, matching with the native vascular tree (Figures 4A–D). The latter was studied on two injected forelimb grafts, which were carefully dissected. Observations showed that the whole vasculature was macroscopically preserved after decellularization, both up to the periosteal and endosteal vascular networks. Moreover, the experiment confirmed that the common pedicle could be individualized in order to create radial or ulnar grafts (Figures 4E,F).

### 3.6 Bone mineral density measurements

**FIGURE 5:**
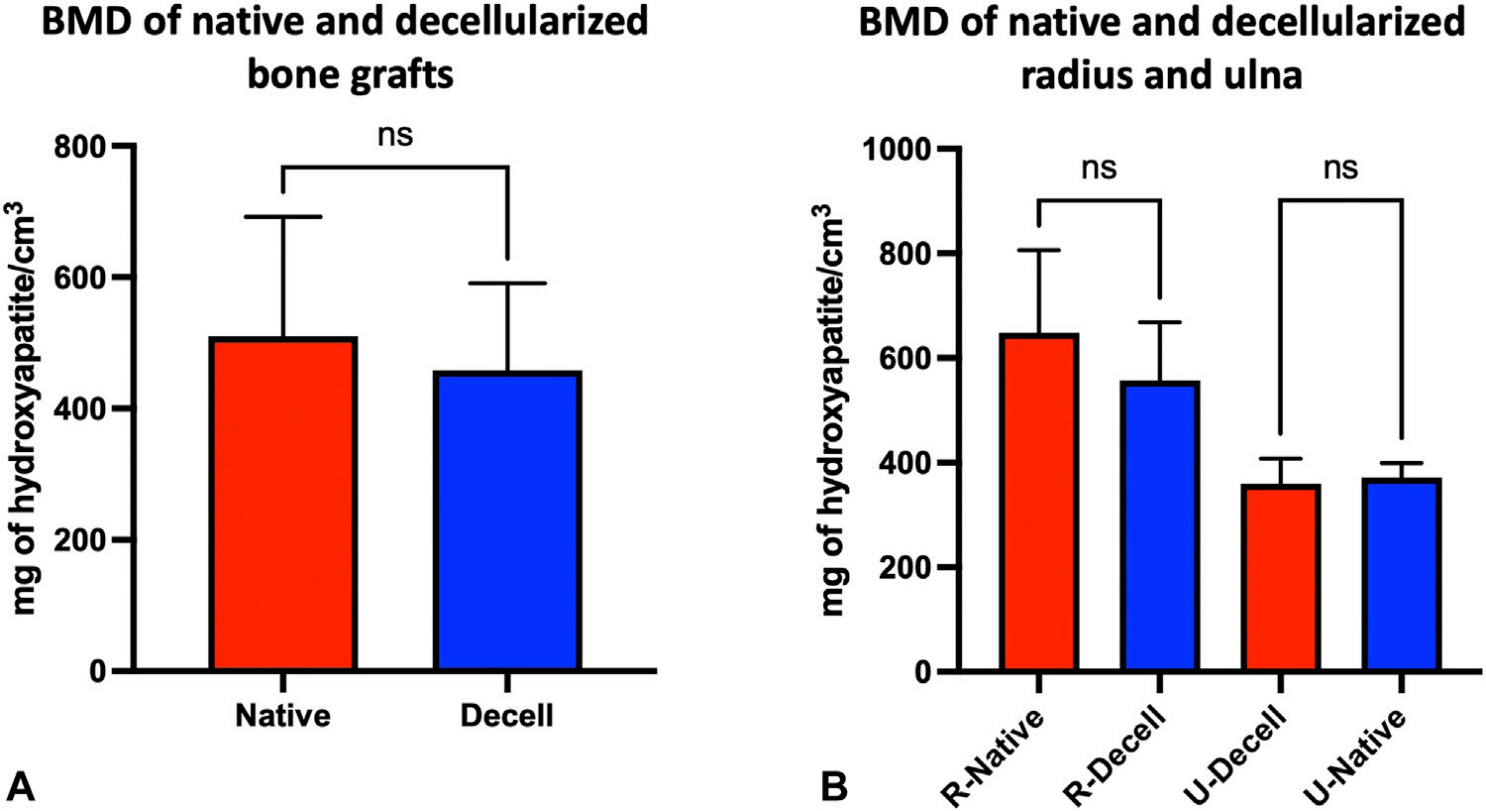
pQCT of vascularized bone grafts. **(A–B)**: Mean value of bone mineral density (BMD) before (native, red) and after decellularization (blue) of vascularized bone grafts (mean of each ulnar and radial BMD values) **(A)**. Specific mean values of radial (R-) and ulnar (U-) BMD before (native, red) and after decellularization (blue) (mean of the three mean values of radius or ulna BMD) **(B)**. BMD values were expressed in mg of hydroxyapatite/cm^3^ ± SD (n = 3 different grafts; *ns* = not significant).

Mean BMD of bone grafts showed no significant difference after decellularization (Nat vs Decell. 509.8 ± 182.5 vs 458.5 ± 132.5 mg of hydroxyapatite/cm^3^, *p* = 0.435, n = 3) (Figure 5A). Regarding the mean BMD values for the radius (R) and ulna (U), no significant difference was observed before and after decellularization. Indeed, the mean BMD for the native radius was 648.2 ± 158.2 mg hydroxyapatite/cm^3^, being 557.2 ± 111.4 mg hydroxyapatite/cm^3^ after decellularization (*p* = 0.75; n = 3). Concerning the ulna, the mean BMD was 371.4 ± 27.62 mg hydroxyapatite/cm^3^ and 359.8 ± 47.91 mg hydroxyapatite/cm^3^ in native and decellularized tissues, respectively (*p* = 0.8148, n = 3) (Figure 5B).

### 3.7 Mechanical testing

**FIGURE 6:**
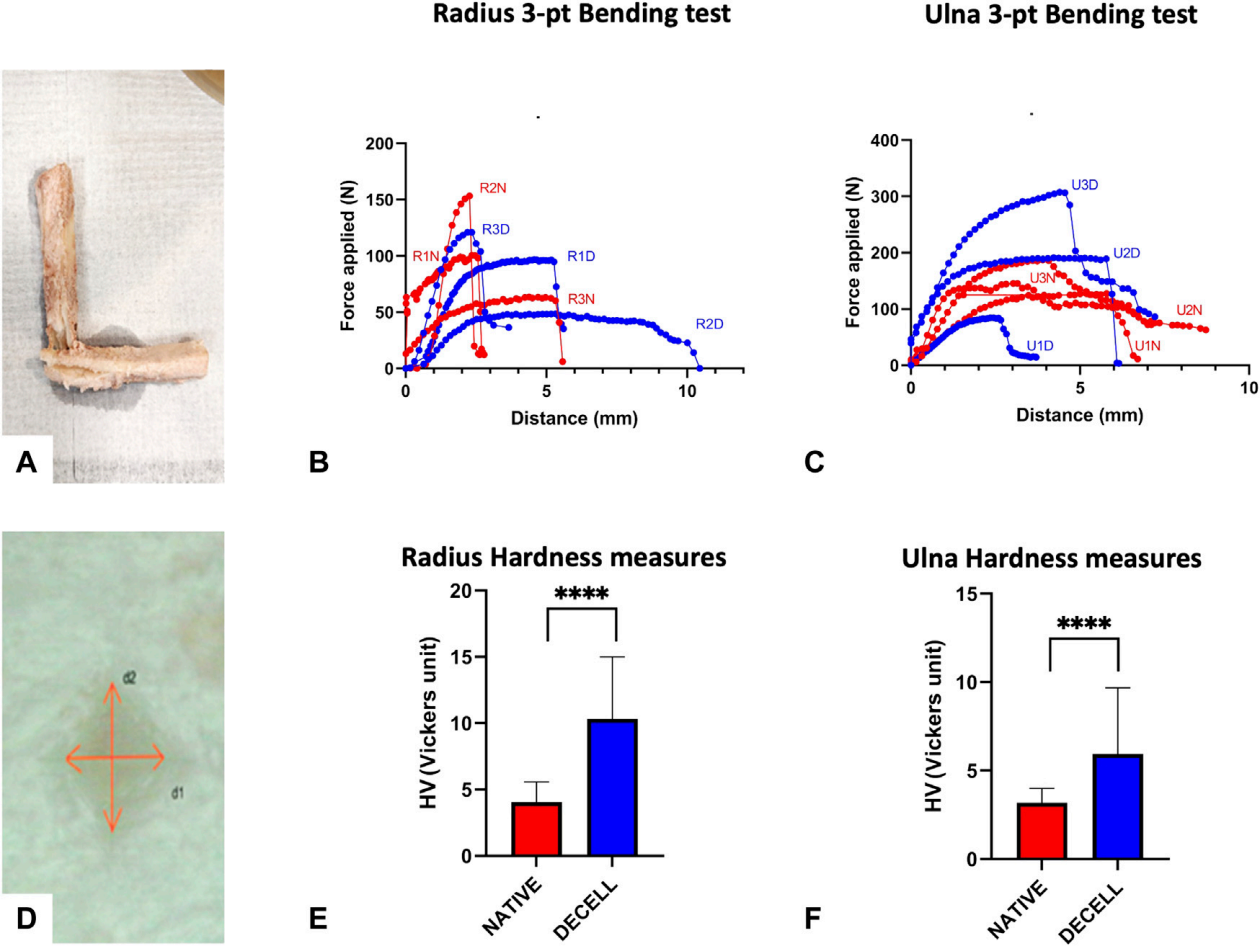
Mechanical Testing bone ECM. **(A–C)**: Bending test performed on radial and ulnar shaft. Visualization of the fracture **(A)** of the rod after successful bending test. Radius **(B)** and ulna **(C)** 3-pt bending curves (red = native, blue = decellularized) achieved while plotting in a graph the force (N) *versus* vertical displacement (mm). **(D–F)** Hardness test on radial and ulnar bone segments. Visualization of the tip print on the mounted sample **(D).** Radius **(E)** and ulna **(F)** hardness measures in native (red) and decellularized bone ECM (blue), Hardness values (HV) were expressed in Vicker units ± SD (n = 3 each; ****p < 0.0001)

The 3-pt bending test revealed that there was a certain tendency towards stiffness decrease for the radial decellularized diaphysis, along with a global stiffness increase for the decellularized ulnar diaphysis. A fracture occurred at 102.3 ± 50.1N/3.3 ± 1.9 mm vs 76.2 ± 41.5N/6.1 ± 3.6 mm for the native *versus* decellularized radial rods (Figure 6B) while a fracture occurred at 137.4 ± 39N/5.4 ± 1.1 mm vs 193.6 ± 113.8N/4.5 ± 1.6 mm for the native *versus* decellularized ulnar rods (Figure 6C). However, given the few tests performed, no statistical analyses were conducted on the 3-pt bending tests for radial and ulnar native and decellularized samples, which were harvested in the same location, *i.e.,* proximal, central, and distal. (Figure 6C). A global and significant increase in the hardness values (HV) was observed following decellularization in both radial and ulnar samples (n = 3 native; 3 decellularized), which turned out to be more significant for the radius samples, *i.e.,* 4.04 vs 10.33 Vicker units for the native *versus* decellularized radial samples (*p* < 0.0001) (Figure 6E) and 3.18 vs 5.94 Vicker units for the native *versus* decellularized ulnar samples (*p* < 0.0001) (Figure 6F).

### 3.8 Cytocompatibility assay: Fibroblastic cell seeding and culture

**FIGURE 7:**
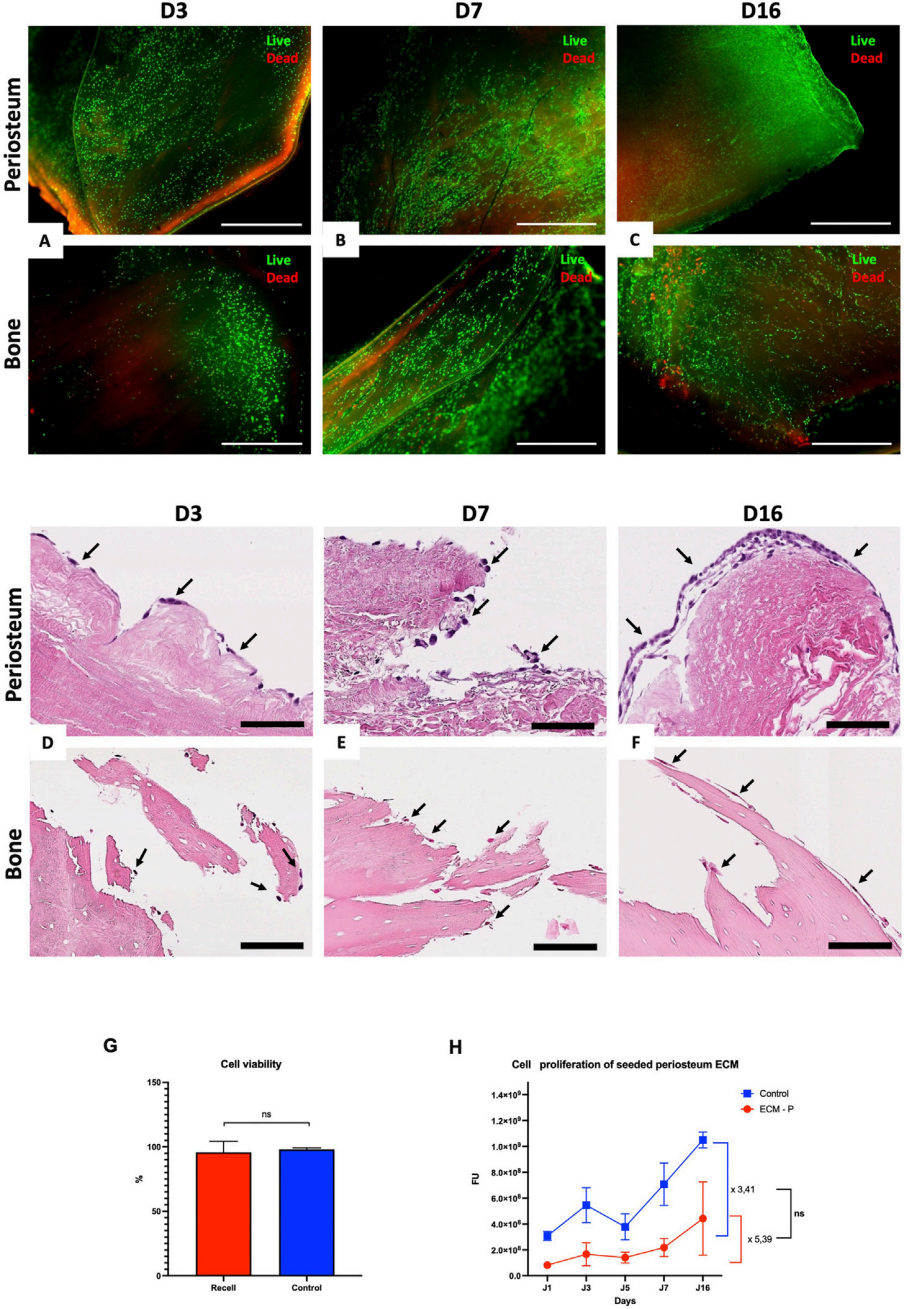
Cytocompatibility study of periosteum and bone ECM with fibroblastic cell line. **(A–C)**: Live/Dead staining of NIH3T3 seeded periosteum ECM (top) and bone ECM (bottom) discs after 3 **(A)**, 7 **(B)** and 16 **(C)** days of culture, respectively, at 2.5 x gross magnification. All scale bars = 1,000 µm. **(D–E)**: H&E staining of NIH-3T3 seeded periosteum ECM (top) and bone ECM (bottom) after 3 **(D)**, 7 **(E)** and 16 **(F)** days of culture, respectively, revealing adherent fibroblasts (black arrows) at 40x gross magnification. All scale bars = 50 µm. **(G)** Cell viability analyzed with four different Live/Dead images at ×2.5 gross magnification of seeded periosteum and culture control wells after seven culture days (n = 3 for each group; *p* = 0.7987, *ns* = non-significant). **(H)** Cell proliferation rate analyzed using Prestoblue Assay during 16 days of culture (n = 3 × 2 ECM; *ns* = non-significant).

Live/Dead staining (Figures 7A–C) and H&E (Figures 7D–F) staining showed adherent and viable cells at the surface of the periosteum and bone ECM discs at Days 3, 7, and 16 of culture, with a higher number of living cells than dead cells. Moreover, H&E staining showed the formation of a continuous cellular layer at the surface of ECM discs, with a specific fibroblast morphology. However, cells exhibited a better adhesion and cohesion along the periosteal ECM fragments than on bone ECM. Cell viability in (% ± SD) quantified on the Live/Dead staining of seeded periosteum after 7 days of culture revealed no difference between ECM and control wells, with a viability of 95.79 ± 2.41% and 98.03 ± 0.35%, respectively (*p* = 0.5512) (Figure 7G). The proliferation rate was evaluated using a PrestoBlue assay, displaying an increase in fluorescence intensity (±SD) from Day 1 to Day 16 for seeded ECM (8.18 × 10^7^ ± 1.92 × 10^7^ to 4.42 × 10^8^ ± 2.83 × 10^8^, *p* = 0.0239) and control wells (3.08 × 10^8^ ± 3.52 × 10^7^ to 1.04 × 10^9^ ± 1.04 × 10^9^, *p* = 0.0286), corresponding to an increase of 5.39-fold and 3.44-fold, respectively, compared to Day 1 (Figure 7H). These findings confirmed the non-cytotoxicity and cytocompatibility of the produced ECM.

### 3.9 pAMSC cell seeding culture and bone-like ECM differentiation

**FIGURE 8:**
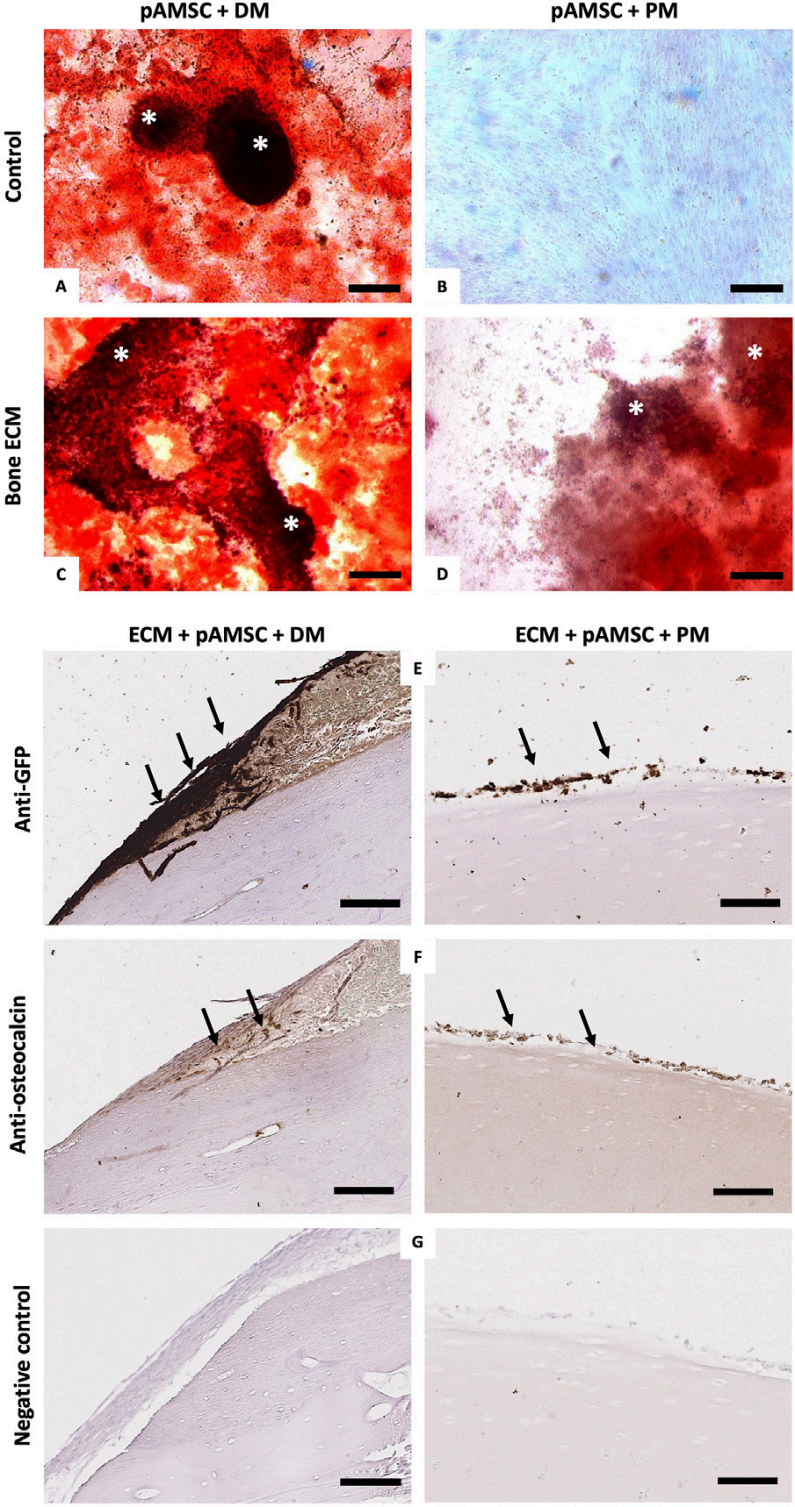
Recellularization of decellularized bone-ECM with pAMSC and osteogenic differentiation. **(A–D)**: Alizarin red staining performed directly in the control culture wells seeded with pAMSC after 7 days of culture with DM **(A)** or PM **(B),** as well as in the culture wells with pAMSC seeded on bone ECM cultured with DM **(C)** or PM **(D)** after 22 culture days showed the formation of calcified micro-nodules (*) in all conditions excepting the seeded cells cultured with PM only. All scale bars = 200 μm. **(E–G)** IHC staining of respectively GFP **(E)**, osteocalcin **(F),** and negative control **(G)** of seeded pAMSC on bone ECM cultured with either DM (left) or PM (right) at 22 days of culture. Black arrows showed the positive osteocalcin staining corresponding to the GFP-pAMSC (All scale bars = 100 µm).

The control culture wells confirmed the osteogenic differentiation of pAMSC with DM after 7 days of culture, reflected by the formation of calcium nodules with Red Alizarin staining (Figure 8A), and the absence of osteogenic differentiation when the pAMSC were cultured with PM only (Figure 8B). In both pAMSC groups that were cultured for 22 days with bone ECM and with either DM or PM, we similarly observed the formation of calcium nodules, thereby confirming that the osteogenic potential of bone ECM was being preserved (Figures 8C,D). The intensity and density of calcium nodules were more significant in the ECM + DM *versus* ECM + PM groups. It was further confirmed by a positive osteocalcin staining detected in both groups of pAMCS cultured on bone ECM with PM or DM, which was performed in parallel by means of GFP staining (Figures 8E–G).

## 4 Discussion

To our knowledge, this is the first study demonstrating the feasibility of attaining a decellularized bone shaft allograft while preserving its vascular pedicle and intrinsic vasculature, harboring its potential for subsequent *in vivo* transplantation. Moreover, this graft was likely able to support new bone formation, after being seeded with the recipient’s mesenchymal stem cells that undergo an ECM- and medium-induced osteogenic differentiation. This original strategy may turn out to be a new path in view of obtaining an upgraded biological substitute for repairing large bone defects. In our initial model design, we chose to harvest the entire porcine forearm including the radius and ulna, rather than only one or the other, in the aim to ensure an optimal preservation of the vasculature with minimal dissection. However, in this study, we demonstrated the option of individualizing either the radius or the ulna on the common interosseous pedicle, which would facilitate the transplantation of a single shaft into a specific critical bone defect. Additionally, based on this model, we demonstrated the decellularization of an entire bone graft, including all its different constitutive tissues. This PDR protocol was adapted based on previous published studies, which were focused on animal and human facial or anatomical subunit tissue engineering (Duisit et al., 2018b; 2018a, 2017; Wüthrich et al., 2020), with significantly increased perfused volumes. Based on the naturally high density of the cortical bone compared with soft tissues mainly face or hand grafts (muscle and fat), we hypothesized that the volume of solvents required would be higher. However, the decellularization and ECM preservation, optimization of decellularization solutions, flows, pressures, and required volumes must still be further explored in the future to be more effective. Our results showed that the DNA amounts of all constitutive tissues of the decellularized vascularized bone graft were below the critical threshold of 50 ng of DNA per mg dry weight, which is considered decellularized and safe after transplantation (Crapo et al., 2011). In addition, the global cell numbers and DNA amounts retrieved from our analyses in native cortical bone and native medulla were very low compared with those from the periosteum or muscle. Each tissue was associated with a significant and evidenced decrease between native and decellularized samples, confirming the efficiency of our protocol, even in tissues with low DNA amounts*.* However, some residual nuclei or cellular debris were observed into the osteocyte lacunae, as previously reported with SDS, SLES and Trypsin/EDTA protocols (Emami et al., 2021). These remnants can be at least partially due to an incomplete washout of cell debris from the bone lacunae, through the very narrow and therefore flow-resistant osteocyte canalicular network in the bone matrix. In addition, this observation could also be explained anatomically by the potential heterogeneity of the forelimb vascular network, supplied by the common interosseous artery which mainly gives rise to a musculo-periosteal vascularization supplied by several small perforating arteries entering the cortical bone through its periosteal surface (Wright and Glowczewskie, 1998). Consequently, an isolated long bone model which has one larger and main nutrient artery, penetrating the medullary cavity and spreading more homogeneously throughout the bone shaft from its endocortical surface should have an improved perfusion, a potentially better decellularization and washing effectiveness. Therefore, it seems to be a better model to study the bone regeneration into a whole vascularized acellular bone graft. Moreover, current preliminary work of our team highlighted a higher efficiency of the cellular removal and washing through the osteocyte lacunae of whole vascularized acellular long bone by using a non-detergent decellularization protocol rather than the classical SDS protocol (Evrard et al.; data not shown, in submission). Nevertheless, the originality of our approach relies in the atraumatic harvesting of the entire intrinsic graft vasculature, which requires optimal preservation of the periosteum and muscular cuff surrounding the bone, by analogy with the clinical practice in bone free flaps harvest (Pederson and Grome, 2019; Bibbo, 2021). Indeed, the alteration of this vascular network during the dissection could be deleterious for the decellularization.

Moreover, our study provided satisfying results with respect to the preservation of ECM architecture and ECM components; these data were similar with those obtained by means of this protocol while using other bioengineered substitutes as reported in the literature, in particular concerning the GAG decrease that we have also reported in this work (Jank et al., 2017, 2015; Duisit et al., 2018b; 2018a, 2017; Gerli et al., 2018; Wüthrich et al., 2020). This point is particularly critical, given that ECM proteins and GAGs have been reported in the literature as being a key point for cell support and further recellularization (Crapo et al., 2011; Peloso et al., 2016). Regarding the biomechanical properties and density acquisitions, we obtained discordant results. Owing to the lack of more samples enabling us to interpret our results in a statistically correct process (including Weibull distribution with two parameters analyses), these issues will be studied in further works to come from our department (Bonney et al., 2011; Setters and Jasiuk, 2014; Coutts et al., 2015; Li et al., 2020). Furthermore, in our cohort, the average age turned out to be very young (8.5 months); therefore, in-growing pigs and fast remodeling bone could have provoked biases in sample comparisons.

Multiple results in our study support the achievement of cytocompatibility and non-cytotoxicity of bone and periosteum ECM, *i.e.,* sterile ECM pieces were obtained *via* PAA and ethanol sterilization (Keane et al., 2015; Balestrini et al., 2016), enabling them to promote cell growth of static seeded NIH-3T3 and pAMSC, despite a longer exposition to the sterilization solution for bone rather than periosteum ECM. Sterilization techniques likely alter ECM, and they are possibly toxic for the seeded cells (Crapo et al., 2011; Tao et al., 2021). Thus, these techniques must be adapted so as to render them less aggressive. Indeed, PAA can alter cell growth, mechanical ECM characteristics, and ECM architecture of from decellularized soft tissues and organs, although other groups did not report such alterations (Moradi et al., 2020). Nevertheless, as previously described, sterilization of decellularized bone scaffolds using PAA or a combination of PAA and SC-CO_2_ (supercritical CO_2_) did not alter ECM’s mechanical and structural characteristics in comparison with commonly employed methods, such as gamma irradiation, electron beam irradiation, and ethylene oxide (Sun et al., 2020; Amirazad et al., 2022). However, by developing a fully vascularized bone graft as a new therapeutic option, this process must be adapted to a large vascularized tissue, either using (Balestrini et al., 2016; Duisit et al., 2018b; 2018a, 2017) or not its vascular pedicle to achieve the sterilization of the whole scaffold (Balestrini et al., 2016; Berger et al., 2020; Liu et al., 2021; Tao et al., 2021; Amirazad et al., 2022), yet without damaging the vascular tree.

All these results confirm the true potential of such matrices for complex bone tissue engineering. Multiple living cells were assessed along the surface of periosteum and bone acellular ECM samples after static seeding, reflecting an ongoing “recellularization”. Moreover, we have demonstrated that the acellular bone ECM obtained displayed the ability to promote *in vitro* osteogenic differentiation of static seeded adipose mesenchymal stem cells, along with new bone-like tissue formation and with a concomitant osteocalcin expression and positive Alizarin Red staining. In addition, this new bone-like tissue could be initiated with the adjunction of either DM or with PM alone. These observations further highlight that several differentiation growth factors were obviously retained in ECM all along the decellularization process. The qualitative nature and quantitative preservation of these factors must indeed be further explored using complementary investigations, including cytokine mapping and immunohistochemical analyses (Mauney et al., 2005, 2004; Schubert et al., 2011). However, in a clinical perspective, the seeding of AMSC and their differentiation in the acellular bone ECM with DM seems more suitable and effective.

Overall, our results appear promising, reflecting a real potential for future *in vivo* reimplantation. However, two main pitfalls regarding the next experimental step must still be emphasized. First, in vascularized solid organ tissue engineering, it has been widely demonstrated that the current main limitations to *in vivo* reimplantation of recellularized as well as vascularized matrices is the restoration of an entire and functional endothelium on the preserved vascular bed in the scaffold (Orlando et al., 2012; Hussein et al., 2020). This regenerated endothelium must be able to ensure a correct physiological interaction between blood cells and platelets, as well as coagulation, adhesion, and aggregation factors which are involved in the coagulation process (Furie and Furie, 1988), in order to restore a long-term perfusable non-thrombogenic vascular bed without edema formation in the scaffold, thus providing oxygen and nutrient supplies to the seeded cells. Despite high efficiency and almost total *in vitro* endothelium restoration in acellular organs showing a physiological vascular flow several days after vascular anastomosis (Uygun et al., 2010; Ren et al., 2015; Hussein et al., 2016; Higashi et al., 2022), this did not prevent significant vascular thrombosis or edema formation from occurring owing to barrier leakiness into the implanted regenerated graft (Petersen et al., 2010; Kitano et al., 2021; Higashi et al., 2022). However, several promising solutions have been developed in organ tissue engineering to attain improved endothelialization. These options comprise a combination of vascular cells, including endothelial, smooth muscle, and pericyte cells (Leuning et al., 2019) or the use of biochemical modifications to the remaining vascular tree ECM (Jackson et al., 2014; Hussein et al., 2016; Devalliere et al., 2018) by means of anti-thrombotic or pro-cell engraftment reagents.

Additionally, *in vivo* reimplantation experiments using a large animal model, first by means of segmental decellularized bone allograft and then using vascularized decellularized bone allograft, should be performed on reliable and replicable animal models of critical-size bone defects. Assessing the quality of bone consolidation through objective outcomes in comparison with current clinical solutions, along with a long-term analysis of the bone graft viability and osteointegration, appears mandatory before considering a potential translation into a human clinical model. Several efforts are currently being performed throughout the global scientific community in order to create reliable bone substitutes with optimal vascularization, osteointegration, and *in vivo* cellular osteogenic differentiation, the one final goal being: the repair of large bone defects.

## 5 Conclusion

We have proposed and demonstrated in this study the feasibility of a new technique based on harvesting fully vascularized bone grafts, which were then decellularized by perfusion in order to obtain a transplantable bone graft “off the shelf”. *In vitro* cell culture results that were collected from our large animal model are promising, as they have demonstrated the initiation of pAMSC osteogenic differentiation on bone-ECM with either standard culture medium (PM) or specific osteogenic culture medium (DM). Further *in vitro* critical experiments are still necessary to assess, during the recellularization step, the correct 3D pattern of cellular distribution in the Haversian system of acellular whole bone graft through vascular seeding by means of a specific perfusion bioreactor. Given this situation, the osteogenic differentiation could be performed following the vascular seeding of mesenchymal stem cells throughout a long *in vitro* cell culture process. Furthermore, the seeding of AMSC and then their oesteogenic differentiation associated with the regeneration of an entire and functional endothelium is undoubtedly necessary to create a non-thrombogenic and viable graft after transplantation, which is capable to undergo a natural bone remodeling that is guided by the recipient, thus allowing for a stable and living osteo-integrated graft.

## Data Availability

The original contributions presented in the study are included in the article/supplementary material, further inquiries can be directed to the corresponding author.

## Abbreviations

CB, cortical bone; CBCT, cone beam computed tomography; DAPI, 4′,6-diamidino-2-phenylindole; DIW, deionized water; DM, differentiation medium; ECM, extracellular matrix; FBS, fetal bovine serum; GAGs, glycosaminoglycans; GFP, green fluorescent protein; H&E, hematoxylin & eosin; HV, hardness values; IHC, immunohistochemistry; M, muscle; MBAS, methylene blue active substances assay; Md, medulla; MT, Masson’s trichrome; P, periosteum; pAMSC, porcine GFP-adipose mesenchymal stem cells; PBS, phosphate-buffered saline; PAA, peracetic acid, PDR, perfusion-decellularization recellularization process; PM, proliferation medium; pQCT, peripheral quantitative computed tomography; P/S, penicillin/streptomycin; RPM, rotation per minute; SC-CO2, supercritical CO2; SD, standard deviation; CO2SD, standard deviation; SDS, sodium dodecyl sulfate; SR, sirius red.
